# Examining the social status, risk factors and lifestyle changes of tuberculosis patients in Sri Lanka during the treatment period: a cross-sectional study

**DOI:** 10.1186/s40248-018-0121-z

**Published:** 2018-04-01

**Authors:** Madapathage Gayan Buddhika Senanayake, Sumudu Indika Wickramasinghe, Sudath Samaraweera, Pubudu De Silva, Sisira Edirippulige

**Affiliations:** 1 Post Graduate Institute of Medicine, University of Colombo, Colombo, -07 Sri Lanka; 2 Medical officer, Ministry of Health, Sri Lanka and PhD candidate, Centre for Online Health, School of Medicine, The University of Queensland, Brisbane, Australia; 3 Deputy Director, National Programme for Tuberculosis Control and Chest Diseases, Public Health Complex, Colombo, -05 Sri Lanka; 4 Consultant Community Physician, Ministry of Health and Indigenous Medicine, Colombo, 10 Sri Lanka; 5 Programme Director (e-Healthcare), Centre for Online Health, The University of Queensland, Brisbane, Australia

**Keywords:** Tuberculosis, Social stigma, Lifestyle, Poverty

## Abstract

**Background:**

Tuberculosis (TB) is a major global health problem, commonly seen in underdeveloped countries. The probability of contracting the disease is significantly higher among the economically vulnerable and the socially disadvantaged. Risk factors associated with TB can also change over time. In the Sri Lankan context, no study has explored how these factors impact patients. Therefore, we aimed to explore social status, associated risk factors and lifestyle changes during the treatment period of TB patients attending a tertiary respiratory center in Colombo, Sri Lanka.

**Methods:**

The descriptive cross-sectional study was conducted in 2011. The study population consisted of diagnosed tuberculosis patients above the age of 15 years. Patient records were retrieved from the TB patient registry for the Colombo district. Systematic sampling was used to identify patients to be invited to the study. An interviewer-administered questionnaire was used for data collection. Data were collected on social status (example, level of education, employment, and income), associated risk factors (example, smoking and alcohol consumption, contact history, narcotic drug use) and lifestyle changes during treatment (example, employment status, social interactions). The analysis included a logistic regression model to explore the association between social status and risk factors.

**Results:**

The total number of patients included in the study was 425. Tuberculosis was found to be strongly prevalent among participants from the lower socio-economic status. It was also common in participants with a low level of education, unemployed, if employed, those who are engaged in unskilled employment and have low levels of income. Risk factors associated with the patients were smoking, alcohol consumptions, narcotic drug use, imprisonment, close contact history with active TB patients and chronic medical conditions. Changes in employment and the reduction of social-interactions were the main lifestyle changes of the participants occurred during the treatment period. The analysis also showed positive correlation between low-level social status and sputum smear infectivity, and use of dangerous drugs. Even after adjusting for confounders, tuberculosis negatively affected social interactions and income levels of participants from the low social status.

**Conclusion:**

Low socio-economic status negatively affected the lifestyle and social interactions of patients during the treatment period. Though competent treatment programs exist in Sri Lanka, it is still important to identify and mitigate risk factors associated with tuberculosis patients. A comprehensive multi-disciplinary approach considering patient lifestyle, and the implications of the disease and treatment on social interactions may strengthen the current preventive strategies.

**Electronic supplementary material:**

The online version of this article (10.1186/s40248-018-0121-z) contains supplementary material, which is available to authorized users.

## Background

Tuberculosis (TB) as an airborne infectious disease is a major global health challenge. Re-emergence of TB has been a concern in recent years [1]. The estimated global incidence of active TB is 10 million with 1.8 million associated deaths each year. One-third of the global population has latent TB [2] with Southeast Asia accounting for 46.5% of the incident cases [3]. Despite Sri Lanka being classified as a low-prevalence country, around 9,000 cases are notified annually with smear-positive disease, at an incident rate of 60% [4].

The probability of contracting the disease is significantly higher among the economically vulnerable and the socially disadvantaged due to increased exposure to active carriers [5]. Key strategies in interruption of transmission of TB include early identification and treatment of patients and tracing their contacts [6]. Management of tuberculosis generally requires continuous treatment for a minimum of 6 months, escalated due to multi-drug resistant forms of the disease [7]. Context-specific reasons to clustering of TB cases in urbanized communities need to be evaluated [8] and novel non-health intervention strategies such as social protection and urban planning are important elements of program planning [9].

The need to adhere to a strict medication regimen often involving multiple drugs affects the lifestyle of a patient leading to changes in their social interactions. The stigma associated with the disease also contributes to lifestyle changes with direct influence on the treatment uptake. Furthermore, education level, employment status, income and household composition are important social and economic factors that influence disease management and its outcomes [10].

Known risk factors associated with TB include positive HIV status, diabetes, smoking, poverty, close contact history and alcohol use [11, 12]. It is important to identify how these risk factors affect TB patients in the Sri Lankan context, as this has not been explored so far. Furthermore, among Sri Lankan TB patients, the impact of treatment on lifestyle, during the treatment period is also not well understood [13]. Any changes to the social status during the treatment period may have an effect on the lifestyle and recovery from the disease. Considering these reasons, we aimed to examine the social status, associated risk factors and lifestyle changes during the treatment period of TB patients attending a tertiary respiratory center in Colombo, Sri Lanka.

## Methods

A descriptive cross-sectional study was conducted in 2011. Data collection was completed during May to December 2011. The social status of patients was assessed using a validated Social Status Index (SSI) tool. It consists of three domains: socioeconomic, asset-ownership and social participation [14]. The risk factors comprised of contact history, medical diseases such as diabetes and bronchial asthma while lifestyle changes included smoking, alcohol intake etc. (Additional file 1 for full survey tool). The lifestyle changes were assessed among diagnosed TB patients who had completed the first 2 months of the treatment regimen. This was done to allow time for patients to develop any lifestyle changes, occurring as a consequence to being diagnosed (and treated) with TB. We also reviewed the associations between social status, risk factors and life style changes.

This study was carried out at the central chest clinic in Colombo, Sri Lanka. This tertiary respiratory center provides preventive and treatment facilities for respiratory diseases to patients from Colombo district, free at the point of delivery when a client is registered. Patients with the respiratory symptoms can be self-referred or referred by other public or private health institutions to the clinic. The study population consisted of all diagnosed tuberculosis patients attending the central chest clinic in Colombo district for a period of 8 months from May to December during 2011. Patients < 15 years of age were excluded as lifestyle changes of children were not independent of their parents. Patients from outside the Colombo district were also excluded.

The overall survey tool consisted of six sections (Additional file 2). Section A includes demographic and general disease-specific data. The SSI is given in section B to D of the survey tool. Section E includes associated risk factors identified from review of literature. Section F contained questions relating to lifestyle changes. Smoking and alcohol consumption was included in both sections E and F, as they are both risk factors and potential lifestyle change elements. The component on influence on lifestyle (section F – Additional file 2) was designed by conducting extensive review of the literature [15–18], key informant interviews and Focus Group Discussions (FGD). Eleven key informant interviews were carried out to understand the lifestyle changes occurring during the course of treatment. Informants included two medical administrators, two respiratory physicians, and three public health specialists from the Colombo central chest clinic, two sociologists and two senior family counselors from the University of Colombo. Later, three FDGs with five TB patients each, were conducted to ascertain social and lifestyle factors associated with the disease. These three FGDs were conducted with patient groups from highly urban, urban and rural areas. Participants consisted of community leaders, retired private and public sector employees, unemployed, housewives, self-employed, slum-dwellers and patients representing the affluent communities.

Upon completion of the first draft of the section on lifestyle changes, consensual validity was assessed by a discussion with eight subject experts. These experts included two public health professionals, two sociologists, two respiratory physicians, a psychologist and a social worker. The second expert team was separate to the previous group of experts who contributed to the streamlining process. Each item in the instrument was assessed for its relevance, appropriateness, and acceptability to the local context, validating agreement to the delivery of conceptual meanings accurately.

The influence of the disease on lifestyle assessed changes to occupation, income level, family relationships and social interactions. Pre-testing of the survey was carried out at Colombo South Teaching Hospital chest clinic on patients who were completing their full course of treatment on the visiting date of the interview. The principal investigator collected data from all participants individually using the survey tool. The participants were selected using systematic sampling, where every third (3^rd^) patient was included from a daily clinic attendance register. Data collection was conducted in a private room in the clinic premises to ensure privacy and confidentiality. Data collected were securely stored with the investigative team.

The primary outcome variable of the study was the social status of participants. All three domains in SSI were considered in five equal quintiles. The quintiles were obtained by running a two-staged Principle Component Analysis (PCA) followed by varimax rotation dividing the sample into five quintiles, which ranged from first quintile (poorest) to fifth quintile (richest). The factor score coefficient of the first factor corresponding to the particular item was considered as the relative weight corresponding to that variable for constructing the composite index for social status (SSI). All items were coded on a binary scale (“0” = absence and “1” = presence). For the questions with multiple responses, each response was considered as a variable. The participants within first, second and third quintiles were considered as ‘low social class’, whereas fourth and fifth quintiles were considered as ‘high social class’.

Analysis of data used the Statistical Package for the Social Sciences 17.0. Two logistic regression (LR) analyses were carried out to identify the level of social status after controlling for the effects of confounders. The model was tested with a backward LR method. Ethical clearance was obtained from Ethical Review Committee of Faculty of Medicine, University of Colombo (EC-11-077).

## Results

A total of 428 Tuberculosis (TB) patients, registered and on treatment at the central chest clinic Colombo were invited to participate in the study. Three patients declined the invitation to participate; a non-response rate of 0.7% (*n* = 3). Demographic and socioeconomic characteristics of the study sample are summarized in Table 1.

**Table 1:** Socio-demographic characteristics of participants (*n* = 425)

Socio -demographic Characteristic	Number	%
Age (in years)		
15–34	109	25.6
25–34	166	39.1
35–44	150	35.3
Ethnicity		
Sinhalese	256	60.2
Tamils	83	19.5
Muslims	80	18.9
Burgher & others	6	1.4
Religion		
Buddhism	244	57.4
Hindu	72	16.9
Islam	78	18.4
Christianity	31	7.3
Current marital Status		
Currently Married	286	67.3
Never Married	98	23.1
Widowed	24	5.6
Divorced/ Separated	17	4.0
Highest level of Education		
Never went to school	48	11.3
Primary education	197	46.3
Secondary education or higher	180	42.4
Daily family income (in USD)		
Less than 2.4 USD (poor income)	73	17.2
2.5–12 USD (low income)	348	81.9
More than 12 USD (middle income)	4	0.9
Employment status		
Employed	224	52.7
Unemployed	201	47.3
Employment Category (*n* = 224)		
Professionals	14	6.3
Clerical and sales workers	34	15.1
Elementary occupations	113	50.5
Others	63	28.1
Social status		
High social status	106	24.9
Low social status	319	75.1

### Social status of participants

Age of included participants ranged between 16 to 90 years with a mean of 47 years (SD - 15.9). Two-thirds of the participants included were males (62.6%; *n* = 266). Equal proportions of the study sample were residing within (49.9%; *n* = 212) and outside (50.1%; *n* = 213) the Colombo Municipal Council (CMC) area. Most of the participants were married (67.3%; *n* = 286) and half of the participants had an education up to primary level [19] (46.3%; *n* = 197). Most of the participants (81.9%; *n* = 348) were earning less than 12 USD per day [20].

Out of the employed participants, 18 (8%) were employed in the public sector and others, from the private sector (*n* = 92, 21%) and in self-employment (*n* = 114, 27%). Amongst the employed, 84% (*n* = 188) were temporarily employed, hence with poor employment security. Only 16.1% (*n* = 36) of included participants were in permanent employment. Out of the employed participants 50.5% were engaged in unskilled occupations [21].

Most participants (75.1%; *n* = 319) belonged to a lower social class, while 24.9% (*n* = 106) were from the higher social status. The mean number of members in the family was 4.7 (SD - 1.67) with a median of five members per family. Families with six members and above consisted of 27.7% (*n* = 118) of the total study sample. The average number of persons sleeping in the same room as the participant (the patient) was 1.7 (SD - 0.83). Thirty households (7.1%) did not have, even a single separate room.

Distribution of housing and selected asset ownership of the study participants across the SSI quintiles is shown in additional file 3. Toilet facilities with ‘flushed to septic tank’ (for safe disposal of sputum and other potentially infectious material) were the commonly used method of the high social class. Fourteen respondents within the poorest quintile did not have any toilet facility. Ten participants (9.4%) from the high social status used electricity for cooking, whilst 158 participants (49.5%) used wood (with potential smoke within the household) as the fuel for cooking from low social strata. An accumulation of assets in households and better housing conditions were seen among those in the highest social status index quintile.

Among participants, 72% (*n* = 306) had been diagnosed with pulmonary TB and the remaining 28% (*n* = 119) with extrapulmonary TB. Sputum smear infectivity (positive smear) was detected from 71.2% of pulmonary TB participants with the rest, identified as sputum smear negative. Tuberculosis peripheral lymphadenopathy was identified in 38 (32.7%) participants.

### Associated risk factors

From the total study sample, 55.1% (*n* = 234) had smoked at least once in their lifetime. Out of the ‘ever-smoked’ participants, 27.4% (*n* = 64) were still continuing to smoke. The prevalence of ever-smoking in the study sample was 55.1 per 100 participants (95% CI: 52.7% -57.5%). The prevalence of ever-alcohol consumption was 54. 6 per 100 in the study sample (95% CI: 52.2% - 57%). The prevalence of dangerous drug use was 12.7 per 100 tuberculosis participants in this sample (95% CI: 11.1% - 14.3%). There were 22 male participants (5.2%; 95% CI: 4.1% - 6.3%) who had been previously imprisoned. The prevalence of confirmed Diabetes Mellitus and Bronchial Asthma were 15.5 per 100 participants (95% CI: 13.7% - 17.3%) and 5.6 per 100 participants (95% CI: 4.5% - 6.7%) respectively.

Univariate analysis (refer to Additional file 4), finds a statistically significant (*p* = 0.002) number of male participants from the lower social status compared to female participants. The level of social status significantly differed between pulmonary and extra-pulmonary TB categories (*p* < 0.001). Participants who used dangerous drugs were significantly at a higher risk of being from a low-level social status than among those who were not using dangerous drugs (p < 0.001). Although participants with a history of imprisonment showed the highest prevalence of low-level social status compared to those not having a history of imprisonment, this difference was not statistically significant (*p* = 0.44).

A logistic regression (LR) analysis, after controlling for the effects of confounders, was carried out to identify the association between demographic, disease-specific and risk factor related factors to the social status of participants. The variables that were significant in the final model tested are presented in Table 2 with their parameter estimates. Out of the 12 independent variables considered in the LR analysis, only 2 variables were retained in the final model (Table 2). These included: sputum smear infectivity, and use of dangerous drugs showing a statistically significant relationship with low-level social status after adjusting for confounders.

**Table 2:** Independent variables of social status and their significance of the final model (*n* = 425)

Independent variable	β	SE (β)	Wald	df	Sig.	Exp (β)	95% CI for Exp (β)	
							Low	Upper
Infectivity: Yes	0.94	0.24	14.6	1	0.000	2.5	1.6	4.1
Use dangerous drugs: Yes	3.03	1.02	8.8	1	0.003	20.6	2.8	152
Constant	0.22	0.17	1.69	1	0.194	1.25		

β – Regression co-efficient; *SE* (β) – standard error of β; *df* = degrees of freedom

Reference categories: Infectivity – No; Use of dangerous drugs – no

Sputum smear infective participants had a 2.5 times higher chance of being from the low-level social status compared to non-infective participants, while dangerous drug users had 20 times higher risk of being from the low-level social status compared to non-dangerous drug users.

### Lifestyle changes

Effects related to lifestyle in 266 (62.6%) participants who had completed the intensive phase (first 2 months) of the treatment were reviewed separately. The employment status had changed for 62.8% (*n* = 167) of the participants, while their income had changed for 52.3% (*n* = 139). Additional file 5 summarizes the association between social status and effects of their lifestyle due to disease. The diagnosis of TB was perceived to be the primary reason for the reduction in frequency of meal intake, frequency of talking with peers or family, and frequency of attending to social gatherings. Six participants (2.3%) had not told their family members that they were on treatment for TB.

Univariate analysis identified the prevalence of low-level of social status to be 83.2% among the study participants who had a change to their employment status post-diagnosis. This was statistically significant (*p* = 0.001) when compared with participants without a post-diagnosis change to their employment status (65.7%). The proportion of those with low-level social status with a perceived negative impact on family life was 82.6%. Only 67.6% of participants from the high-level social status strata, perceived such negative influence on their family life post-diagnosis; a statistically significant association (*p* = 0.005). Among participants with changes to smoking habits post-diagnosis, 79.7% were from the low-level social status category. Among the participants who had not changed their smoking practices, this proportion was 73.9%; *p* = 0.27 (Refer to Additional file 5).

A second logistic regression model was developed to study the association of low-level social status with factors affecting lifestyle. Table 3 summarizes the adjusted OR (aOR), their 95% confidence interval and the statistical significance of the affected lifestyle factors adjusted for confounders. Out of the 10 independent variables considered in the LR analysis, only two variables were retained in the final model. After adjusting for confounders, change in income level (aOR = 4.06, 95% CI: 2.1–7.75); and influence to social gatherings (aOR = 3.1, 95% CI: 1.5–6.3) were the statistically significant factors that influenced a participant’s lifestyle when low-level of social status was considered.

**Table 3:** Independent variables of social status and their significance of the final model describing the effects of lifestyle (*n* = 266)

Independent variable	β	SE (β)	Wald	df	Sig.	Exp (β)	95% CI for Exp (β)	
							Low	Upper
Change income level: yes	1.4	0.33	18.1	1	0.000	4.06	2.1	7.75
Influence to social gathering: Yes	1.13	0.36	10.0	1	0.002	3.1	1.5	6.3
Constant	−0.29	0.33	0.76	1	0.38	0.75		

β – Regression co-efficient; *SE* (β) – standard error of β; *df* = degrees of freedom

Reference categories: change income level – no; influence to social gathering – no;

## Discussion

This is the first study conducted in Sri Lanka describing the social status, associated risk factors and lifestyle changes during the treatment period of TB patients attending a tertiary respiratory center in Colombo. The study found a higher proportion of the patients with TB to be from the low social strata. They were more likely to have a lower educational level, be unemployed or if employed, to be engaged in unskilled occupations. Similarly, Squire and others [22] have previously identified poverty as significant determinant in TB control.

Associated risk factors identified from the study include smoking, alcohol consumption, dangerous drug use, imprisonment, close contact history with active patients and chronic medical conditions. Smoking is an important preventable risk factor for TB [23]. In a prospective cohort study by Jee et al. [24], current smokers were found to be having a higher probability of developing TB compared to ex-smokers. Furthermore, the risk was positively correlated with the number of cigarettes smoked daily. The prevalence of ever-smoking in this study was 55.1%, hence it is possible smoking had a role to play in the disease process itself. Furthermore, smoking increases the risk of recurrence of TB even after an effective course of treatment [25], highlighting the need for a tightened smoking cessation program in TB control [26]. Evidence shows that effective cessation of smoking has a positive impact on TB mortality rates [27]. Time-based modeling by Lin et al. [28] showed a gradual and complete cessation of smoking of the global population by the year 2033, to reduce the TB disease burden by 14% - 52% if the DOTS coverage was maintained at 80%. The findings from our study show 12% of the respondents (*n* = 32) who had ever-smoked still continuing to smoke despite their disease status and treatment regimen, highlighting the need for comprehensive smoking cessation interventions to be integrated into future programs in Sri Lanka [29].

Majority of the study sample (54.6%) were ever-alcohol consumers with 76 patients (32.8%) continuing to consume alcohol even subsequent to the diagnosis. Similar to smoking, alcohol consumption is a well-identified risk factor for TB [30, 31], causing delays to care seeking itself [32]. The high prevalence of alcohol consumption among our study sample may be linked with the high proportion of persons in unskilled occupations and a lower level of education. Managing TB among alcohol users requires rigorous patient support and social security measures. A multidisciplinary approach is needed for health and social care systems to gain better results [33].

Dangerous drug use was seen among 12.7% of the study sample. Addictive substances reduce the immune mechanisms of the body leading to challenges in screening, diagnosis, and treatment of TB [34]. Drug use also causes poor adherence to treatment, increasing drug interactions, and the emergence of drug-resistant TB - a major obstacle in TB control [35]. Therefore, identifying structural and organizational barriers and forming a systematically coordinated approach with close monitoring is necessary for TB prevention and control interventions [36].

Kelly [37] describes how active TB patients perceive themselves as a disease vector, leading to isolation and neglect as they attempt to keep the disease a secret. In this study, 7 participants (1.6%) stated that they were living alone and six participants (2.3%) had not told their family members that they were on treatment for TB. Being secretive about the disease prevents successful contact-tracing affecting further case detection. The causes of the stigma associated with TB include fear of infection, social beliefs, and practices, blaming and shaming the TB patients, association with HIV and self-stigmatization [38]. Family as the smallest unit of society is directly influenced by TB due to the psychological impact. Macq et al. [39] describe the severe psychological trauma with TB, due to poor self-confidence and depression stemming from not involving the family in the care process.

This study revealed that 62.8% (*n* = 167) of participants have changed their job due to TB, while 52.3% (*n* = 139) had changes to their income level. Needham et al. [40] found high absenteeism among patients and in some instances patients halting employment altogether as a result of the disease. The resultant loss of income negatively affects the individual and the dependent family, most likely driving them into poverty [41]. Social security through government interventions is important for low-income families once a diagnosis is made during this period of insecurity.

The main strengths of this study was that it was carried out in a real clinical setting with an appropriate sample size to interpret the results. This is also the first study to explore social status and lifestyle changes of TB patients in the Sri Lankan context. The use of the social status index, which is a validated tool among Sri Lankan adults, to assess social aspects is another strength of this study. The descriptive cross-sectional study design was used to gather information from individuals, but it is not suitable to identify causal relationships. Although face, content and consensual validity were ascertained for the section on life style changes of the survey tool, it was not statistically validated prior to use. The results of this study are based on data gathered at one center (a tertiary care center in Colombo). Therefore, the findings are most likely not generalizable.

## Conclusions

Social and economic aspects have a significant impact on patients with TB. This study found that TB was strongly associated with the low socioeconomic status. It was also found that socially disadvantaged groups report more instances of negative lifestyle changes, subsequent to diagnosis and initiation of treatment. The study showed that the majority of TB patients in Sri Lanka encounter socio-economic problems such as unemployment, low income and poor social interactions after being diagnosed with TB. Therefore, it is important to pay greater attention to social status and lifestyle changes during the treatment period of TB patients as part of disease management process. A broad multidisciplinary approach should be established with smoking, alcohol, and dangerous-drug cessation interventions included. Addressing socio-economic issues may help achieving positive outcomes related to treatment and may also assist in Sri Lanka achieving tuberculosis elimination targets in the future.

## Additional files

Additional file 1: A Study on Social status and associated factors of tuberculosis patients attending to Central Chest Clinic Colombo and the influence of the disease on their life style. (DOCX 288 kb)

Additional file 2: Contents of the survey tool. (DOCX 14 kb)

Additional file 3: Housing and asset ownership across the Social Status Index quintiles in the study population (*n* = 425). (DOCX 16 kb)

Additional file 4: Level of social status correlated with demographic, disease-specific and risk factor related characteristics of the study population (*n* = 425). (DOCX 18 kb)

Additional file 5: Level of social status by effects to the lifestyle of the study population (*n* = 266). (DOCX 18 kb)

## Abbreviations

CMC: Colombo Municipal Council
DOTS: Directly Observed Treatment Short course
FGD: Focus Group Discussion
HIV: Human Immuno-deficiency Virus
LR: Logistic Regression
OR: Odds Ratio
PCA: Principle Component Analysis
SD: Standard Deviation
SSI: Social Status Index
TB: Tuberculosis
