# Supplementary material for: Examining the social status, risk factors and lifestyle changes of tuberculosis patients in Sri Lanka during the treatment period: a cross-sectional study
Source: Multidiscip Respir Med. 2018 Apr 1;13:9. doi: 10.1186/s40248-018-0121-z (PMC5878935; doi:10.1186/s40248-018-0121-z)
Supplement: Supplementary file 1 — A Study on Social status and associated factors of tuberculosis patients attending to Central Chest Clinic Colombo and the influence of the disease on their life style. (DOCX 288 kb) [file 40248_2018_121_MOESM1_ESM.docx]

**Confidential**  Serial Number: ……………

A Study on Social status and associated factors of tuberculosis patients attending to Central Chest Clinic Colombo and the influence of the disease on their life style.

Please introduce yourself. Explain the objectives of the study. Explain how he/she was selected for the study. Ensure confidentiality of the information. Obtain inform verbal consent.

**Questionnaire**

**Section A: Demographic information**  District TB Number: …………..

1. What is your Date of Birth? Day Month Year

|  |  |  |
| --- | --- | --- |

1. How old are you? Age ……………………….. (in years on the last birthday)
2. Sex – 1. Male 2. Female
3. What is the type of disease do you have? (*If your answer is “a”, go to question 5; otherwise 6.)*

a. Pulmonary TB

b. Extra pulmonary TB Site …………………

1. What is the type of sputum did you have at the beginning?

a. Sputum smear positive

b. Sputum smear negative

1. What is your treatment category?

a. New d. Treatment after failure

b. Relapse e. Treatment after default

c. Transfer in f. Others (specify)………………..

1. What is the date of commencement of treatment? ………………….
2. What is your residential Address? - ………………….………………………

………………………………………….

1. How long have you been in that residence? - …………………………..
2. Ethnicity – 1. Sinhalese 2..Tamil

3. Muslim 4. Burger

5. Others (Please Specify) ……………………….

1. Religion – 1. Buddhism 2. Hindu

3. Islam 4. Christian

5. Others (Please specify)…………………………

1. Marital Status – 1. Married 2. Never married

3. Divorced 4. Separated

5. Widowed 6. Living together

7. Others (Please specify) ………………………

1. With whom are you living? (can tick more than one option)
2. With spouse 2. With children
3. With siblings 4. With grand parents

5. With parents 6. With other relatives

7. With non- relatives 8. Others (Specify) …………

14. How many members are there in your family? .....................

15. What is the relationship with the head of the family?

1. Head 2. Spouse 3. Father

4. Mother 5.Son 4.Daughter

7. Brother 8. Sister 9.No relationship

10. Other relationship (specify)……………………..

16. What is your highest educational achievement?

1. No schooling 2. Grade 1 to 5 3. Grade 6 to 8 4. Grade 9 to 11 5. Passed GCE O/L 6. Passed GCE A/L

7. University 8. Others (Specify)………………….

17. What is your employment status?

1. Employed 2. Unemployed

If employed,

18.1 What type of employer are you?

1. Government 2. Private

18.2 What is the nature of your occupation?

1. Permanent 2. Temporary

18.3 Are you entitled to pension?

1. Yes 2. No

18.4 Please state the place that you are working? …………………………………………..

18.5 What is your designation? ……………………………………….

19. What is the health care institution you usually visit for treatments?

1. Government hospital 2. Private hospital

3. Central Dispensary 4. General Practice

5. Others (specify) ……………………………

20. What is the distance to the above mentioned heath care institution from your residence?

………………….. Km.

**Section B: Housing conditions**

21. What is the tenure of your house?

1. Owned by a member of the household 2. Rent free

3. Rent/ Lease 4. Encroached

5. Official quarters 6. Others (specify)………………….

22. What is the type of your house?

1. Detached house 2. Line house

3. Slum 4. Flat

5. Refugee camp 6. No settlement

23. What materials are used for the following in your house?

**Wall Roof Floor**

Bricks Tiles Cement

Cabook Asbestos Concrete

Cement blocks Metal sheets Tiles/ Terrazzo

Stones Concrete Granite

Pressed soil blocks Cadjan Wood planks

Mud Palmyrah Dung/earth

Plank Straw Sand

Metal sheet Metal sheets Bamboo

Cadjan ………… Polished wood

Other ………….

24.1 Do you have air conditioned rooms at your home?

1. Yes 2. No

If yes;

24.2 Please state how many rooms are air conditioned? ……..

25. Do you have a swimming pool at your home?

1. Yes 2. No

**Part C - Household amenities**

26. State regarding the sanitation facilities available at your home?

1. Exclusive for the household

2. Having, but sharing with another household

3. Not having, but sharing with other house hold

4. Common/ public toilet

5. Not using a toilet

6. Others (specify) …………………….

27. What is the type of toilet do you have at your home?

1. Water seal 2. Pour flush

3. Pit latrine 4. Bucket latrine

5. Not available 6. Others (specify)…………

28. State regarding the availability of water at your home?

1. Exclusively for the household.

2. Having, but sharing with another household

3. Not having, but sharing with another household.

4. Common/public

5. Other (specify)……………….

29. What is the source of the drinking water?

1. Protected well (within premises) 5. Tap within premises

2. Protected well (outside the premises) 6. Tap outside premises

3. Unprotected well 7. Distributing vehicle

4. Tube well 8. Others (specify) ………………

30. What is the principal type of lighting in your home?

1. Kerosene 2. Electricity

3. Solar power 4. Others (specify)…………….

31. What is the principal type of cooking fuel used in your home?

1. Firewood 2. LP gas/ Bio gas

3. Kerosene 4. Electricity

5. Saw dust / paddy husk 5. Other (specify) ………………

32. What is the place of cooking in your home?

1. Inside the house 2. Temporary hut

3. in a separate building 4. Outdoors

33. How many rooms are used for sleeping? …………………

Availability of equipment’s

Does your household have the followings?

34. Telephone

a. Land line c. CDMA

b. Mobile d. Other (Specify) ………………………..

35. Internet

a. Broadband b. Dial up

36. Vehicle

a. Bicycle e. Van

b. Motorcycle f. Tractor

c. Three wheeler g. Lorry

d. Car h. Bus

i. Others (specify) ………………………….

37. Electric Items

a. Radio c. Refrigerator e. Computer

b. Television d. Microwave

38. Animal

a. Cattle c. Poultry

b. Goat d. Pig

39. Please be kind enough to give the monthly income details of the family

a. Income from the employment : Rs…………..

b. Income from rent/lease of land or other assets : Rs……………

c. Income from selling products : Rs……………

d. Income by providing private services : Rs……………

e. Other sources of income : Rs……………

f. Total income : Rs……………

40. What is your contribution to the family income?

1. No contribution 4. Contribution is more than half

2. Little contribution 5. Sole contribution

3. Almost half contribution

**Part D– Social participation** (social status questionnaire, A.P de silva.2011)

41.1.1 Has the head of the household (HoH)/ family member in your house made a donation (money/ equipment) to your village, society of the village or temple during the past one year period? 1. Yes 2. No

*If ‘Yes’;* 41.1.2. What is the cost of the donation: Rs………………..

41.1.3 If the donor is a family member, then what is his/ her relationship to the head of the household?

1. Parent 2. Child 3. Sibling

4. Other (specify) …………..

42.2.1. Are you/ family member in your house is a member of a village/ work place societies?

1. Yes 2. No - skip to 42.3

42.2.2. If ‘Yes’; Name the society and your/ family member’s designation in each of them.

Information on participation in societies

| Relationship to HOH | Name of the society | Designation |
| --- | --- | --- |
|  |  |  |
|  |  |  |
|  |  |  |

42.3.1 Is the head of the household/ family member, in your house is a member of a political party? 1. Yes 2. No

42.3.2 If ‘Yes’, then is the HoH/ family member in your house a member of the following councils?

1. Urban council 2. Pradeshiya sabha

3. Provincial council 4. Parliment

42.3.3 If a family member in your house is a member of the above council, then what is his/her relationship to head of the household?

1. Parent 2. Child

3. Sibling 4. Other (specify) ……………………………

**Part E – Associated factors for TB**

43.1 Have you ever smoked? Yes No

[*If yes, go to Question 41.2. Otherwise go to Question 42.1]*

43.2 Do you smoke now? – Yes No

If yes,

43.3 What is the type?

a. cigarette d. pipe

b. cigars e. Other (specify) …………

b. beedi

43.4 What is the daily consumption?

a. 1 – 5 c. 10-20

b. 6- 10 d. >20

44.1 Have you ever consumed alcohol? Yes No

[*If yes, go to Question 42.2; otherwise go to Question 43.1]*

44. 2 Do you consume alcohol now? Yes No

44.3 What is the type of alcohol you usually drink?

a. Arrack d. beer

b. Toddy e. others (specify)…………..

c. Kassippu

44.4 What is the frequency?

a. Once a week d. 1 – 4 times a day

b. 2-6 times a week e. >5 times /day

c. Once a day

45.1 Have you ever used any dangerous drugs such as heroin or cannabis (Ganja)?

Yes No

*(If yes; go to question 43.2, otherwise question 44.1)*

45.2 What is the type of drug/s that you have used?

a. Heroin c. Others (specify)…………

b. Cannabis (Ganja)

45.3 What is the frequency of drug use?

a. Once a week d. 1- 4 times a day

b. 2-6 times a week e.> 5 times a day

c. Once a day

Close contact history

46.1 Do you have any of your close associates (relatives, neighbors, collogue at workplace) who have taken anti TB drugs for the last 2 years or who was diagnosed to be having TB?

Yes No

*(If yes; go to question 44.2, otherwise 45)*

46.2 How was he /she associated with you?

a. Household c. same workplace

b. Neighbors d. Other (specify) ……………………….

46.3 What is the reason for you to state he/she had TB?

a. On suspicion c. was told by others

b. Told by the patient d. aware he/she took treatment

46.4 If at household, what is the relationship?

a. Spouse b. son c. daughter

c. brother d. sister f. mother

e. father g. other (specify)…………………

47. Have you ever imprisoned for more than 24 hours during the past 2 years?

a. Yes b. No

Are you suffering from any of the following diseases?

48.1 Diabetes Mellitus – Yes No

48.2.a If yes, confirmed - (diagnosis card available)

48.2. b Unconfirmed - (subject says so, but no documentary evidence available)

49.1 Bronchial Asthma – Yes No

49.2.a If yes, Confirmed – (if diagnosis card available)

49.2.b Unconfirmed – (subject says so, but no documentary evidence available.)

50.1 Any other chronic health problems (kidney disease, malignancy, organ transplant)

Yes No

*(If yes; go to 48.2, otherwise 49)*

50.2 What is the chronic health problem that you are suffering?

a. Chronic Kidney disease. c. organ transplant

b. Malignancy d. Others (specify) ………………..

50.3 Do you have documents to prove it?

a. Diagnosis card available – confirmed

b. Subject says so, no documentary evidence available – unconfirmed

**Component 2**

**Note: *Only if the patient has completed the first two months of treatment.***

**Part F – Influence of the disease on the life style**

51. What has happened to your smoking habit?

a. Not relevant, non-smoker d. intended to reduce/stop in near future

b. Reduced the frequency e. increased smoking

c. Continue with the same phase f. completely Stopped

g. others (specify) …………………………..

52. What happened to your alcohol intake?

a. Non alcoholic d. intended to reduce/stop in near future

b. Reduced the frequency e. increased alcohol intake

c. Continue with the same phase f. completely Stopped

g. other (specify) …………………

53. Is there any change in your dietary habits?

a. no change in diet d. lost the interest for certain foods

c. change in dietary habits e. Improved dietary intake

b. lost the appetite significantly

54.1 What happened to your job after the diagnosis of TB?

a. No change in the job e. lost the job

b. Job changed but in the same work place f. on leave

c. Change the job in different place g. other (specify)………..

d. Voluntarily stopped doing the job

54.2 If there is any change, is it because you got TB?

Yes No

55.1 What happened to your income level after the diagnosis of TB?

a. income not changed c. c. Lost the total income

b. lost the income partially d. income improved.

55.2 If there is any change, is it due to the TB?

Yes No

Regarding the interaction with your family members:

56.1 Have you ever told to your family members about your disease status?

Yes No

56.2 If yes, what happened to your family relationship?

a. Living as usual

b. Went to a different home

c. Went to a different home living with others(not with family members)

d. Living alone

e. Family members left home

f. Living in the same home but separated from the family

g. Others (specify)…………………………..

57.1 If you are married what has happened to your marital status?

a. No change c. separated

b. Divorced d. Other (specify)……………

57.2 If there is any change, is it due to the TB?

Yes No

What has happened to your interactions with your family members (talking, taking meals, watching TV, playing together, etc)?

58.1 Frequency of taking meals with your family members-

a. Decreased than usual c. increased than usual

b. Not changed (continue as usual)

58.2 If there is a change, do you think that it is due to the disease status?

Yes No

59.1 Frequency of talking with your family members-

a. Decreased than usual c. increased than usual

b. Not changed (continue as usual)

59.2 Do you think that it is due to the disease status?

Yes No

60.1 Frequency of attending the recreational activities (playing games, watching TV) at home-

a. Decreased than usual c. increased than usual

b. Not changed (continue as usual)

60.2 Do you think that it is due to the disease status?

Yes No

Interaction with the society

61.1 Frequency of attending to the social gatherings, meetings as before- a. Decreased than usual c. increased than usual

b. Not changed (continue as usual)

61.2 If changed, Do you think that it is mainly due to the disease?

Yes No

61.3 If it is decreased than usual; what are the reasons for it? (Can have multiple responses)

a. People rejected me

b. People did not rejected me but I afraid so

c. I was advised by the doctor to do so

d. I was asked by the family to do so

e. I am not healthy enough to continue as usual

f. Other reason (specify)………………………………..
