# Supplementary material for: Examining the social status, risk factors and lifestyle changes of tuberculosis patients in Sri Lanka during the treatment period: a cross-sectional study
Source: Multidiscip Respir Med. 2018 Apr 1;13:9. doi: 10.1186/s40248-018-0121-z (PMC5878935; doi:10.1186/s40248-018-0121-z)
Supplement: Supplementary file 2 — Contents of the survey tool. (DOCX 14 kb) [file 40248_2018_121_MOESM2_ESM.docx]

**Additional file 2 Table S4: Contents of the survey tool**

| **Section** | | **Content** |
| --- | --- | --- |
| Section A | Demographic data | Sex, age, marital status, level of education, occupation, religion, ethnicity |
|  | General data of the disease | Type of the disease (pulmonary or extra pulmonary), sputum smear is positive or not, treatment category and date of commencement of treatment. |
| Section B | Housing conditions | Tenure and type of house, materials used for roof, floor and wall, Other facilities (swimming pool and air condition) |
| Section C | Household amenities | Water and sanitation facilities, source of energy, commodities available, financial status of the family. |
| Section D | Social participation | Donations, participate in village/work place societies, involvement with politics |
| Section E | Risk factors for TB | History of smoking, alcohol intake, narcotic drug use, close contact history with TB, presence of chronic diseases such as Diabetes mellitus, Bronchial Asthma, Kidney diseases and malignancies. |
| Section F | Influence of the disease on the life style | Influence to the food habits, marital status, employment status, income level and risk behaviours (alcohol, smoking and drug use), social behaviours and interaction with their family and interaction with the society (attending to social gatherings and meetings) |
