# Supplementary material for: Examining the social status, risk factors and lifestyle changes of tuberculosis patients in Sri Lanka during the treatment period: a cross-sectional study
Source: Multidiscip Respir Med. 2018 Apr 1;13:9. doi: 10.1186/s40248-018-0121-z (PMC5878935; doi:10.1186/s40248-018-0121-z)
Supplement: Supplementary file 3 — Housing and asset ownership across the Social Status Index quintiles in the study population (n = 425). (DOCX 16 kb) [file 40248_2018_121_MOESM3_ESM.docx]

**Additional file 3:**

**Housing and asset ownership across the Social Status Index quintiles in the study population (n=425)**

| **Selected asset item** | **Social status Index quintiles** | | | | |
| --- | --- | --- | --- | --- | --- |
|  | **1**  **Poorest** | **2**  **Second poorest** | **3**  **Middle** | **4**  **Second richest** | **5**  **Richest** |
| **Toilet facility** |  |  |  |  |  |
| Flushed to septic tank | 17 (8.2) | 32 (15.5) | 52 (25.1) | 48 (23.2) | 58 (28.0) |
| No facility | 14 (100.0) | 0 (0) | 0 (0) | 0 (0) | 0 (0) |
| **Floor type** |  |  |  |  |  |
| Cement/ concrete | 20 (8.5) | 46 (19.5) | 64 (27.1) | 38 (16.1) | 68 (28.8) |
| Terrazzo/ granite | 0 (0) | 0 (0) | 1 (25.0) | 1 (25.0) | 2 (50.0) |
| Wood plank | 24 () | 2 () | 0 (0) | 0 (0) | 0 (0) |
| **Roof type** |  |  |  |  |  |
| Finished asbestos | 30 (12.7) | 40 (17.0) | 60 (25.4) | 45 (19.1) | 61 (25.8) |
| Metal sheets | 64 (94.1) | 3 (4.4) | 1 (1.5) | 0 (0) | 0 (0) |
| **Exterior wall** |  |  |  |  |  |
| Bricks | 33 (14.6) | 39 (17.3) | 48 (21.2) | 37 (16.4) | 69 (30.5) |
| Plank | 33 (97.1) | 1 (2.9) | 0 (0) | 0 (0) | 0 (0) |
| **Fuel for cooking** |  |  |  |  |  |
| Electricity | 0 (0) | 0 (0) | 0 (0) | 3 (30.0) | 7 (70.0) |
| LP gas | 17 (9.7) | 25 (14.2) | 38 (21.6) | 44 (25.0) | 52 (29.5) |
| Wood | 73 (46.2) | 52 (32.9) | 33 (20.9) | 0 (0) | 0 (0) |
| **Facility** |  |  |  |  |  |
| Land phone | 3 (1.8) | 14 (8.6) | 40 (24.6) | 46 (28.2) | 60 (36.8) |
| Refrigerator | 3 (1.6) | 24 (13.1) | 50 (27.4) | 52 (28.4) | 54 (29.5) |
| Microwave | 0 (0) | 0 (0) | 0 (0) | 14 (35.9) | 25 (64.1) |
| Computer | 0 (0) | 0 (0) | 2 (3.6) | 21 (38.2) | 32 (58.2) |
| Internet | 0 (0) | 0 (0) | 1 (3.8) | 6 (23.1) | 19 (73.1) |
| **Vehicles** |  |  |  |  |  |
| Car/van/ lorry | 0 (0) | 0 (0) | 0 (0) | 7 (41.2) | 10 (58.8) |
| Motor cycle | 2 (2.9) | 7 (10.3) | 12 (17.6) | 27 (39.7) | 20 (29.5) |
| Bicycle | 7 (15.6) | 14 (31.1) | 20 (44.4) | 4 (8.9) | 0 (0) |
