# Supplementary material for: Examining the social status, risk factors and lifestyle changes of tuberculosis patients in Sri Lanka during the treatment period: a cross-sectional study
Source: Multidiscip Respir Med. 2018 Apr 1;13:9. doi: 10.1186/s40248-018-0121-z (PMC5878935; doi:10.1186/s40248-018-0121-z)
Supplement: Supplementary file 4 — Level of social status correlated with demographic, disease-specific and risk factor related characteristics of the study population (n = 425). (DOCX 18 kb) [file 40248_2018_121_MOESM4_ESM.docx]

**Additional file 4:**

**Level of social status correlated with demographic, disease-specific and risk factor related characteristics of the study population (n=425)**

| **Characteristic** | **Social status** | | | | **Statistics** | | |
| --- | --- | --- | --- | --- | --- | --- | --- |
|  | **Low** | | **High** | | **Odds ratio** | **95% CI** | **Significance** |
|  | **No** | **%** | **No** | **%** |  |  |  |
| **Sex** |  |  |  |  |  |  |  |
| Male | 213 | 80.1 | 53 | 19.9 | 2.00 | 1.3– 3.1 | x^2^=9.6,df=1, **p=0.002** |
| Female | 106 | 66.7 | 53 | 33.3 | 1.00 |  |  |
| **~~Ethnicity~~** |  |  |  |  |  |  |  |
| ~~Non-Sinhalese~~ | ~~142~~ | ~~84.0~~ | ~~27~~ | ~~16.0~~ | ~~2.30~~ | ~~1.4-3.8~~ | ~~x~~^~~2~~^~~=12,df=1,~~ **~~p=0.001~~** |
| ~~Sinhalese~~ | ~~177~~ | ~~69.1~~ | ~~79~~ | ~~30.9~~ | ~~1.00~~ |  |  |
| **~~Religion~~** |  |  |  |  |  |  |  |
| ~~Non-Buddhist~~ | ~~149~~ | ~~82.3~~ | ~~32~~ | ~~17.7~~ | ~~2.00~~ | ~~1.3-3.1~~ | ~~x~~^~~2~~^~~=8.9,df=1,~~ **~~p=0.003~~** |
| ~~Buddhist~~ | ~~170~~ | ~~69.7~~ | ~~74~~ | ~~30.3~~ | ~~1.00~~ |  |  |
| **TB category** |  |  |  |  |  |  |  |
| Pulmonary | 248 | 81.0 | 58 | 19.0 | 2.90 | 1.8-4.6 | x^2^=21,df=1, **p=0.000** |
| Extra-pulmonary | 71 | 59.7 | 48 | 40.3 | 1.00 |  |  |
| **Infectivity** |  |  |  |  |  |  |  |
| Sputum positive | 184 | 84.4 | 34 | 15.6 | 2.90 | 1.8-4.6 | x^2^=21,df=1, **p=0.001** |
| Sputum negative | 135 | 65.2 | 72 | 34.8 | 1.00 |  |  |
| **Current smoking** |  |  |  |  |  |  |  |
| Yes | 58 | 90.6 | 6 | 9.4 | 3.70 | 1.5-8.8 | x^2^=9.7,df=1, **p=0.002** |
| No | 261 | 72.3 | 100 | 27.7 | 1.00 |  |  |
| **Dangerous drug use** |  |  |  |  |  |  |  |
| Yes | 53 | 98.1 | 1 | 1.9 | 20.9 | 2.8-153 | x^2^=16,df=1, **p=0.000** |
| No | 266 | 71.7 | 105 | 28.3 | 1.00 |  |  |
| **History of imprisonment** |  |  |  |  |  |  |  |
| Yes | 21 | 95.5 | 1 | 4.5 | 7.40 | 0.98-55 | x^2^=5.1,df=1, p=0.44 |
| No | 298 | 73.9 | 105 | 26.1 | 1.00 |  |  |
| **Living alone** |  |  |  |  |  |  |  |
| Yes | 6 | 85.7 | 1 | 14.3 | 2.00 | 0.2-17 | x^2^=0.4,df=1, p=0.24 |
| No | 313 | 74.9 | 105 | 25.1 | 1.00 |  |  |
