# Supplementary material for: Examining the social status, risk factors and lifestyle changes of tuberculosis patients in Sri Lanka during the treatment period: a cross-sectional study
Source: Multidiscip Respir Med. 2018 Apr 1;13:9. doi: 10.1186/s40248-018-0121-z (PMC5878935; doi:10.1186/s40248-018-0121-z)
Supplement: Supplementary file 5 — Level of social status by effects to the lifestyle of the study population (n = 266). (DOCX 18 kb) [file 40248_2018_121_MOESM5_ESM.docx]

**Additional file 5:**

**Level of social status by effects to the lifestyle of the study population (n=266)**

| **Characteristic** | **Social status** | | | | **Statistics** | | |
| --- | --- | --- | --- | --- | --- | --- | --- |
|  | **Low** | | **High** | | **Odds ratio** | **95% CI** | **Significance** |
|  | **No** | **%** | **No** | **%** |  |  |  |
| **Change of job** |  |  |  |  |  |  |  |
| Yes | 139 | 83.2 | 28 | 16.8 | 2.60 | 1.5-4.6 | x^2^=10.7,df=1, **p=0.001** |
| No | 65 | 65.7 | 34 | 34.3 | 1.00 |  |  |
| **Change income level** |  |  |  |  |  |  |  |
| Yes | 123 | 88.5 | 16 | 11.5 | 4.40 | 2.3-8.2 | x^2^=23,df=1, **p=0.000** |
| No | 81 | 63.8 | 46 | 36.2 | 1.00 |  |  |
| **Influence to family life** |  |  |  |  |  |  |  |
| Yes | 133 | 82.6 | 28 | 17.4 | 2.30 | 1.3-4.1 | x^2^=7.9,df=1, **p=0.005** |
| No | 71 | 67.6 | 34 | 32.4 | 1.00 |  |  |
| **Influence to marital status** |  |  |  |  |  |  |  |
| Yes | 7 | 70.0 | 3 | 30.0 | 0.70 | 0.2-2.8 | x^2^=0.26,df=1, p=0.6 |
| No | 197 | 77.0 | 59 | 23.0 | 1.00 |  |  |
| **Influence to dietary habits** |  |  |  |  |  |  |  |
| Yes | 143 | 80.8 | 34 | 19.2 | 1.90 | 1.1-3.5 | x^2^=4.9,df=1, **p=0.026** |
| No | 61 | 68.5 | 28 | 31.5 | 1.00 |  |  |
| **Influence to talking** |  |  |  |  |  |  |  |
| Yes | 163 | 81.5 | 37 | 18.5 | 2.70 | 1.5-5.0 | x^2^=10.4,df=1, **p=0.001** |
| No | 41 | 62.1 | 25 | 37.9 | 1.00 |  |  |
| **Influence to recreational activities** |  |  |  |  |  |  |  |
| Yes | 136 | 82.4 | 29 | 17.6 | 2.30 | 1.3-4.1 | x^2^=7.9,df=1, **p=0.005** |
| No | 68 | 67.3 | 33 | 32.7 | 1.00 |  |  |
| **Influence to attending social gatherings** |  |  |  |  |  |  |  |
| Yes | 178 | 81.3 | 41 | 18.7 | 3.5 | 1.8-6.8 | x^2^=14.6,df=1, **p=0.000** |
| No | 26 | 55.3 | 21 | 44.7 | 1.00 |  |  |
| **Changes to smoking habit** |  |  |  |  |  |  |  |
| Yes | 102 | 79.7 | 26 | 20.3 | 1.4 | 0.8-2.5 | x^2^=1.2,df=1, p=0.27 |
| No | 102 | 73.9 | 36 | 26.1 | 1.00 |  |  |
| **Changes to alcohol habit** |  |  |  |  |  |  |  |
| Yes | 104 | 80.0 | 26 | 20.0 | 1.4 | 0.8-2.6 | x^2^=1.6,df=1, p=0.21 |
| No | 100 | 73.5 | 36 | 26.5 | 1.00 |  |  |
